# The role of thyroid function on the occurrence and psychopathological exacerbation of delusional disorders: Two case studies and review of recent works.

**DOI:** 10.1192/j.eurpsy.2023.2221

**Published:** 2023-07-19

**Authors:** A. Guàrdia, A. González-Rodríguez, L. Lafuente, M. Natividad, E. Izquierdo, E. Roman, J. A. Monreal

**Affiliations:** 1 Psychiatry, Hospital Universitari Mutua de Terrassa, Terrassa, Spain

## Abstract

**Introduction:**

Primary hypothyroidism has been extensively associated with the presence of depressive symptoms in major depressive and bipolar disorders; however, the association between psychosis and hypothyroidism has received less attention.

**Objectives:**

We aimed to present two cases of patients with delusional disorder (DD) and hypothyroidism and review studies focused on this association.

**Methods:**

(1)Two case studies of DD patients. (2)Narrative review on the association of hypothyroidism and psychosis by using PubMed database (2000-August 2022). Search terms: [Hypothyroidism AND (psychosis or delusional disorder)].

**Results:**

*Two case-studies.* Case A: 58 year-old woman with DD who presented a worsening of psychotic symptoms in association with the occurrence of newly diagnosed hypothyroidism. Risperidone 1mg daily was initiated. A combination of levothyroxine 100 mcg/day and paroxetine 20mg/day was started. Case B: 51 year-old DD women with remission of delusional symptoms, who presented occurrence of depressive symptoms and panic attacks with agoraphobia. Olanzapine 5 mg/day and venlafaxine 225 mg/day were started combined with levothyroxine 75 mcg/day.

*Review*: From a total of 159 records, 52 studies described an association between psychosis and hypothyroidism. Most of the studies are focused on the Myxedema madness, treatment of psychosis with comorbid hypothyroidism, and the role of thyroid function on emerging psychoses. Others: intellectual disability, epilepsy, psychosis, asthma, diabetes and heart failure. Genetic associations of Xq13 gene, encoding for nuclear receptors of thyroid receptors, with psychosis.

**Conclusions:**

Many questions pertaining to DD and thyroid function remain unanswered. Treatment of hormonal comorbidities may be associated with a clinical improvement of psychotic symptoms.

**Disclosure of Interest:**

None Declared

